# Long‐term humoral immunity decline in hemodialysis patients following severe acute respiratory syndrome coronavirus 2 vaccination: A cohort study

**DOI:** 10.1002/hsr2.854

**Published:** 2022-10-03

**Authors:** Eibhlin Goggins, Binu Sharma, Jennie Z. Ma, Jitendra Gautam, Brendan Bowman

**Affiliations:** ^1^ Division of Nephrology University of Virginia School of Medicine Charlottesville Virginia USA; ^2^ Public Health Sciences, University of Virginia School of Medicine Charlottesville Virginia USA

**Keywords:** antibody, COVID‐19 vaccination, end‐stage kidney disease, hemodialysis, humoral immunity, SARS‐CoV‐2

## Abstract

**Background and Aims:**

Dialysis patients are extremely vulnerable to severe acute respiratory syndrome coronavirus 2 (SARS‐CoV‐2) infection with high rates of hospitalization and mortality rates. In January 2021, the University of Virginia Dialysis Program initiated a program‐wide vaccination campaign to administer the Pfizer BioNTech messenger RNA SARS‐CoV‐2 (BNT162b2) vaccine. The aim of this study was to characterize the long‐term time‐dependent decline in humoral immunity in hemodialysis patients.

**Methods:**

A prospective cohort study measuring serial monthly semiquantitative IgG antibody levels to the SARS‐CoV‐2 spike protein receptor binding domain in fully vaccinated in‐center hemodialysis patients. Samples were collected monthly and tested for anti‐SARS‐CoV‐2 antibodies against the anti‐spike S1 domain for 2–6 months post full vaccination. Results were presented as internationally harmonized binding antibody units (BAU/ml). To analyze the change in antibody levels over time, a linear mixed model with random intercept and random slope was used for longitudinal antibody levels. A multivariable model was used to estimate the slope of antibody levels by adjusting for selected patient characteristics. Based on the estimated intercepts and slopes for each subject from the unadjusted model, 10‐month antibody levels were projected.

**Results:**

The mean baseline antibody level was 647.59 BAU/ml and 87.88% (29/33) of patients were considered qualitatively positive. Two patients were negative at baseline and an additional two had borderline results. Patient antibody levels declined at an adjusted average rate of 31% per month. At 6 months postvaccination, 40% of patients remaining in the cohort possessed either negative or borderline IgG antibody levels. Projecting future antibody levels suggests that 65% of the cohort will progress to borderline or negative antibody levels at 10 months post full vaccination.

**Conclusion:**

The long‐term vaccine response following vaccination with the BNT162b2 in hemodialysis patients was characterized. Our data add to the limited pool of data in this patient population and emphasize the critical need for vaccine boosters.

## 1 INTRODUCTION

Among patients with end‐stage kidney disease (ESKD), morbidity and mortality from infection with severe acute respiratory syndrome coronavirus 2 (SARS‐CoV‐2) are high.^1^ Dialysis patients are at high risk of infection with SARS‐CoV‐2 due to impaired humoral and cellular immune function and high rates of underlying comorbidities. Initial trials of the Pfizer‐BioNTech COVID‐19 vaccine (BNT162b2) demonstrated robust antibody responses in the general population; however, patients with ESKD were excluded from these studies.^2^

Data regarding SARS‐CoV‐2 vaccine humoral response in dialysis patients has recently been reviewed.^3^ Briefly, 80%–90% of patients with ESKD attain detectable IgG antibodies to the spike protein receptor‐binding domain component of SARS‐CoV‐2.^4^, ^5^, ^6^ These rates, while impressive, remain lower than those observed in the general population. Dialysis patients also attain lower antibody levels in response to messenger RNA vaccines compared to healthy controls.^7^ Despite the majority of dialysis patients attaining a positive antibody response, the strength and long‐term durability of humoral immunity following coronavirus disease 2019 (COVID‐19) vaccine regimens is incompletely understood.

In January 2021, the University of Virginia began a program‐wide vaccination campaign for dialysis patients exclusively using BNT162b2 (courtesy of the Virginia Department of Health) and achieved an 80% vaccination rate among prevalent dialysis patients.^8^ From this cohort, a subset of patients was selected to prospectively study serial antibody levels from 2 to 6 months following full vaccination. Here, the results of serial monthly antibody levels, slope of antibody decline, and qualitative population loss of detectable humoral antibody response in this selected subset are reported.

## 2 METHODS

### 2.1 Study population

Sixty‐nine patients undergoing in‐center hemodialysis were confirmed as fully vaccinated at the sole University of Virginia study site/dialysis center. Of these, 35 adults (>18 years) were enrolled in this study. The sample size was based on pragmatic considerations of sample volume processing capacity. All participants received two doses of the BNT162b2 vaccine between January and February 2021. Patients dialyzing for acute kidney injury and those with active infection or suspected SARS‐CoV‐2 infection requiring isolation were excluded at enrollment (Supporting Information: Figure 1).

### 2.2 Sample collection and assessment

Samples were obtained on a monthly basis beginning at a mean of 9.1 weeks post full vaccination (defined as >14 days following second immunization) on designated collection dates for each dialysis shift (Monday‐Wednesday‐Friday or Tuesday‐Thursday‐Saturday). A 10 ml EDTA tube was collected from each patient's dialysis blood line during dialysis treatment, stored in a designated research refrigerator, and processed within 8 h of initial collection. Tubes were centrifuged at 3000 rpm (1620 rcf) for 10 min in the swing bucket rotor (S4180) at 4°C using a Beckman GS‐15R centrifuge. Plasma obtained was stored at −80°C in 0.5 ml aliquots until further analysis.

All monthly EDTA plasma samples were tested for anti‐SARS‐CoV‐2 antibodies against the anti‐spike S1 domain using the commercially available Anti‐SARS‐CoV‐2 QuantiVac ELISA (IgG) from Euroimmun (EUROIMMUN US, Inc.). Assays were run and results were interpreted as per the manufacturer's guidelines. Samples above detection limits were rerun with further dilution (1:5 or 1:10) in the sample buffer as recommended by the manufacturer. Based on the manufacturer's recommendation, the final test results were presented as the internationally harmonized binding antibody units (BAU/ml).^9^ BAU/ml was obtained by multiplying the relative unit (RU/ml) by a factor of 3.2. Final test results were considered negative for BAU/ml (<25.6), borderline for BAU/ml (≥25.6 and <35.2), and positive for BAU/ml (≥35.2).^9^

To assess for undiagnosed prior infection and confirm reported histories of prior infection, the Bio‐Rad Platelia SARS‐CoV‐2 Total Ab assay (Bio‐Rad Laboratories, Inc.) was used for the qualitative detection of total antibodies (IgM/IgG/IgA) to SARS‐CoV‐2 nucleocapsid protein. Testing was run from EDTA plasma and limited to each patient's initial sample only. Recombinant SARS nucleocapsid protein is used in the assay to capture total antibodies in a one‐step antigen capture format, followed by detection.

### 2.3 Data collection

Demographic data including age, sex, race/ethnicity, and body mass index (BMI) and clinical data, including comorbidities, use of immune suppressive medication, history of malignancy, and history of transplantation were obtained from the Electronic Health Record (Table 1). Clinical information including dialysis vintage was obtained from the dialysis‐specific electronic medical record system. The mean age was 62.0 years; 51.43% were women and 60% were African American. The mean dialysis vintage was ~4.5 years. Sixty percent of patients were diagnosed with diabetes mellitus and 42.86% were obese (BMI > 30); 25.71% had a history of malignancy and 17.14% had a history of solid organ transplant.

**Table 1:** Patient characteristics by prior COVID‐19 infection and immune suppression

	Prior COVID‐19 infection		Immune suppression		
Characteristics	No (*N* = 29)	Yes (*N* = 6)	No (*N* = 26)	Yes (*N* = 9)	Overall (*N* = 35)
Age	62.55 ± 11.20	59.33 ± 11.00	64.54 ± 9.360	54.67 ± 12.87	62.00 ± 11.07
Female	15 (51.7%)	3 (50.0%)	13 (50.0%)	5 (55.6%)	18 (51.4%)
Race					
African American	16 (55.2%)	5 (83.3%)	17 (65.4%)	4 (44.4%)	21 (60.0%)
White	13 (44.8%)	0 (0%)	8 (30.8%)	5 (55.6%)	13 (37.1%)
Asian	0 (0%)	1 (16.7%)	1 (3.8%)	0 (0%)	1 (2.9%)
Etiology ESRD					
DM	9 (31.0%)	1 (16.7%)	8 (30.8%)	2 (22.2%)	10 (28.6%)
GN	4 (13.8%)	0 (0%)	2 (7.7%)	2 (22.2%)	4 (11.4%)
HTN	5 (17.2%)	2 (33.3%)	5 (19.2%)	2 (22.2%)	7 ((20.0%)
Other	11 (37.9%)	3 (50.0%)	11 (42.3%)	3 (33.3%)	14 (40.0%)
Dialysis vintage (years), median [Q1–Q3]	4.48 [1.64, 8.90]	4.18 [3.04, 10.17]	4.78 [2.06, 8.33]	4.20 [0.79, 10.83]	4.48 [1.84, 9.87]
Access type					
AVF	16 (55.2%)	2 (33.3%)	14 (53.8%)	4 (44.4%)	18 (51.4%)
CVC	13 (44.8%)	4 (66.7%)	12 (46.2%)	5 (55.6%)	17 (48.6%)
Solid organ transplants	5 (17.2%)	1 (16.7%)	2 (6.16)	4 (45.4%)	6(17.1%)
Cancer history	8 (72.4%)	4 (66.7%)	5 (19.2%)	5 (55.6%)	10 (28.6%)
Comorbidities					
DM	17 (58.6%)	4 (66.7%)	16 (61.5%)	5 (55.6%)	21 (60.0%)
HTN	29 (100%)	6 (100%)	26 (100%)	9 (100%)	35 (100%)
CVA	8 (27.6%)	0 (0%)	6 (23.1%)	2 (22.2%)	8 (22.9%)
COPD	4 (13.8%)	0 (0%)	4 (15.4%)	0 (0%)	4 (11.4%)
CHF	13 (44.8%)	4 (66.7%)	12 (46.2%)	5 (55.6%)	17 (48.6%)
MI	6 (20.7%)	2 (33.3%)	6 (23.1%)	2 (22.2%)	8 (22.9%)
PAD	3 (10.3%)	0 (0%)	1 (3.8%)	2 (22.2%)	3 (8.6%)
Obesity (BMI > 30)	12 (41.4%)	2 (33.3%)	11(42.3%)	3(33.3%)	14(42.86%)
Immune suppression	8 (27.6%)	1 (16.7%)	_	_	9 (25.7%)
Prior COVID‐19 infection	_	_	5 (19.2%)	1 (11.1%)	6 (17.1%)
BAU/ml (month 2)	371.0 ± 407.6	1892 ± 866.3	741.0 ± 827.2	398.6 ± 604.4	647.6 ± 779.2
BAU/ml (month 6)	66.99 ± 66.79	710.4 ± 450.1	222.6 ± 348.9	60.71 ± 75.3	177.9 ± 306.2

Abbreviations: AVF, arteriovenous fistula; BAU, binding antibody unit; CHF, chronic heart failure; COPD, chronic obstructive pulmonary disease; COVID‐19, coronavirus disease 2019; CVA, central venous access; CVC, central venous catheter; DM, diabetes mellitus; ESRD, end‐stage renal disease; GN, glomerulonephritis; HTN, hypertension; MI, myocardial infarction; PAD, peripheral artery disease.

Prior COVID‐19 infection information was collected from a designated tracking file in the dialysis unit and verified with SARS‐CoV‐2 nucleocapsid protein assay results.

### 2.4 Statistical methods

Data were summarized as mean and standard deviation or median (25th, 75th percentiles) for continuous variables and as frequency and percentage for categorical variables. The main objective was to estimate the slope of antibody level decline from the time of full immunization to 6 months post full immunization. To analyze the change in antibody levels over time, a linear mixed model with a random slope and random intercept was used for longitudinal antibody levels to account for patient‐specific changes and variation over the entire follow‐up period. The antibody level was natural log transformed for its skewed distribution before the analysis. Univariate models were used to test the association of the trajectories of antibody levels with patient characteristics including prior COVID‐19 infection, immune suppression, gender, age, race, Charlson comorbidity index (CCI), and access type. The interactive effect of prior COVID‐19 infection and immune suppression variable with time were also tested. A multivariable model was used to estimate the slope of antibody levels by adjusting for selected patient characteristics, including age, gender, prior COVID‐19 infection, and immune suppression because of the small sample sizes. In addition, based on the estimated intercepts and slopes for each subject from the unadjusted model, a 10‐month antibody level was projected and plotted in a spaghetti graph versus the observed value. *p* < 0.05 was considered significant. All analyses were performed using software R (version 3.6.3).

## 3 RESULTS

Table 1 provides the clinical characteristics of all study subjects. Three participants previously tested positive for COVID‐19 and three additional prior infection cases were identified using SARS‐CoV‐2 nucleocapsid protein assay results, yielding 17% of the study population with prior infection. Nine subjects were defined as immune suppressed at baseline based on current immune suppressive medication use or predisposing medical condition. One patient had a prior infection and was also categorized as immune suppressed. Over the course of the study, one patient withdrew consent, another received a successful transplant, and three patients died from causes unrelated to SARS‐CoV‐2 infection.

A total of 153 samples were collected from 35 patients. Out of 35, 25 (71%) patients completed all five sample collections. Baseline (i.e., month 2) spike protein IgG levels in BAU/ml are presented in Table 1. The mean baseline antibody level was 647.59 BAU/ml, and 87.88% (29/33) of patients were considered qualitatively positive based on cutoffs provided by the manufacturer (Figure 2). Two patients were negative at baseline and an additional two had borderline results, yielding an 88% overall initial positive response. Of the initial four borderline or negative subjects, two were categorized as immune suppressed and another had a history of malignancy, consistent with prior studies.

**Figure 1:**
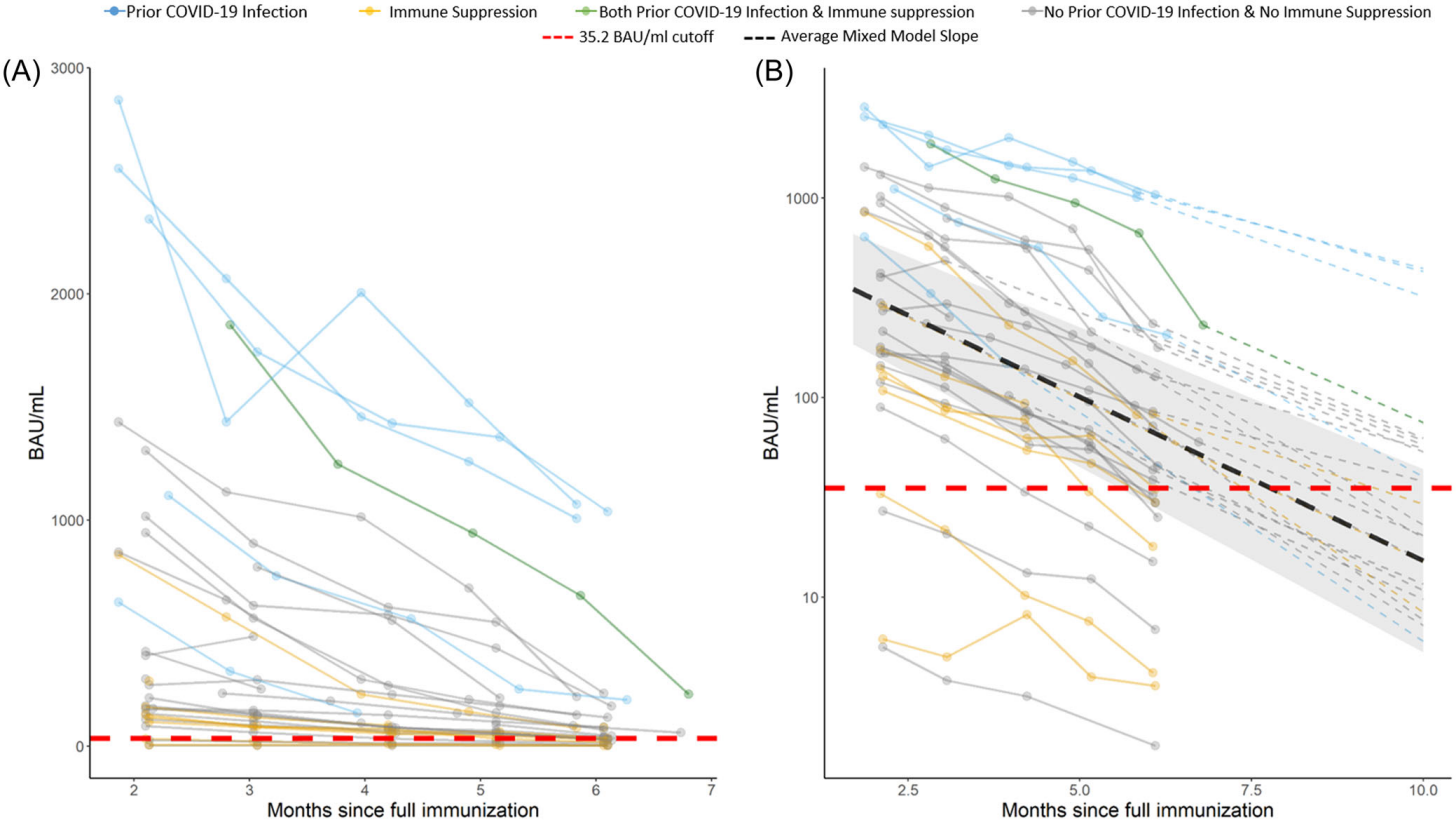
(A) Observed antibody level of SARS‐CoV‐2, the lines are colored by prior COVID‐19 infection and immune suppression status. (B) Logarithmic scale (*y*‐axis) prediction graph on individuals post 10 months since full vaccination. The dark lines are observed values and the dotted lines are predicted values (dashed line not shown for subjects who are “negative” or “borderline” at month 6). Individual intercept and slope estimated from an unadjusted linear mixed model were used for prediction. The cutoff for borderline/negative antibody level is 35.2 (red dashed line). COVID‐19, coronavirus disease 2019; SARS‐CoV‐2, severe acute respiratory syndrome coronavirus 2.

At 3, 4, 5, and 6 months following full vaccination, the average antibody levels fell to 491.4, 365.6, 302.0, and 177.9 BAU/ml, respectively (Figure 1 and Supporting Information: Figure 2). As expected, the antibody levels on the log scale significantly declined over time (*p* < 0.05). Further, the unadjusted results (Table 2) show that prior COVID‐19 infection was significantly associated with attained antibody level (*p* < 0.001), but that immune suppression was not (*p* = 0.12). On average, patients with prior COVID‐19 infection had nine times higher antibody levels than those without. Age, gender, dialysis vintage, CCI, immune suppression, and access type were not significantly associated with antibody level. Race was significantly associated; however, the relationship was spurious as five of six patients with prior infection were African American (Table 1). The interactive effect of prior COVID‐19 infection and immune suppression with time was not significant (Table 2), suggesting that the log‐linear decay of antibody levels in these patients was similar. The decline in the antibody level by notable clinical characteristics is detailed in Supporting Information: Table 1.

**Table 2:** Univariate and multivariate results from linear mixed model of log antibody level

	Univariate analysis		Multivariable analysis	
Effects	Estimates (95% CI)	*p* value	Estimates (95% CI)	*p* value
Time (per month)	−0.38 (−0.43, −0.32)	<0.001	−0.37 (−0.43, −0.32)	<0.001
Age (per year)	−0.02 (−0.07, 0.2)	0.283	−0.03 (−0.08, −0.00)	0.075
Male	0.41 (−0.61, 1.44)	0.423	0.27 (−0.51, 1.06)	0.502
Race				
No African American (reference)				
African American	1.03 (0.05, 2.01)	0.044		
Dialysis vintage				
≥5 years (reference)				
<5years	−0.43 (−1.43, 0.56)	0.398		
Charlson comorbidity index (per unit)	−0.03 (−0.23, 0.17)	0.773		
Access				
AVF (reference)				
CVC	−0.23 (−1.2, 0.80)	0.651		
Immune suppression (yes)	−0.90 (−2.01, 0.20)	0.120	−1.11 (−2.07, −0.14)	0.038
Prior COVID‐19 infection (yes)	2.19 (1.09, 3.31)	<0.001	1.97 (0.95, 3.00)	0.001
Time × prior COVID‐19 infection	0.04 (−0.09, 0.17)	0.581		
Time × immune suppression	−0.00 (−0.12, 0.11)	0.959		

Abbreviations: AVF, arteriovenous fistula; CI, confidence interval; COVID‐19, coronavirus disease 2019; CVC, central venous catheter.

The adjusted multivariable linear mixed model included time, age, gender, prior COVID‐19 infection, and immune suppression. The race was not included as noted above. After adjustment, time, prior COVID‐19 infection, and immune suppression were significantly associated (*p* < 0.05) and age was marginally associated (*p* = 0.075) with the trajectory of antibody level (Table 2). Keeping all other variables constant, the antibody level per month decayed by an average of 31%. Older patients experienced greater decay in the antibody levels, at an additional 4% decline for 1‐year increment in age. Immune suppressed patients, on average, had a 65% lower antibody level compared to patients without immune suppression and patients with prior COVID‐19 infection had five times higher antibody levels than infection naïve patients (Table 2).

**Figure 2:**
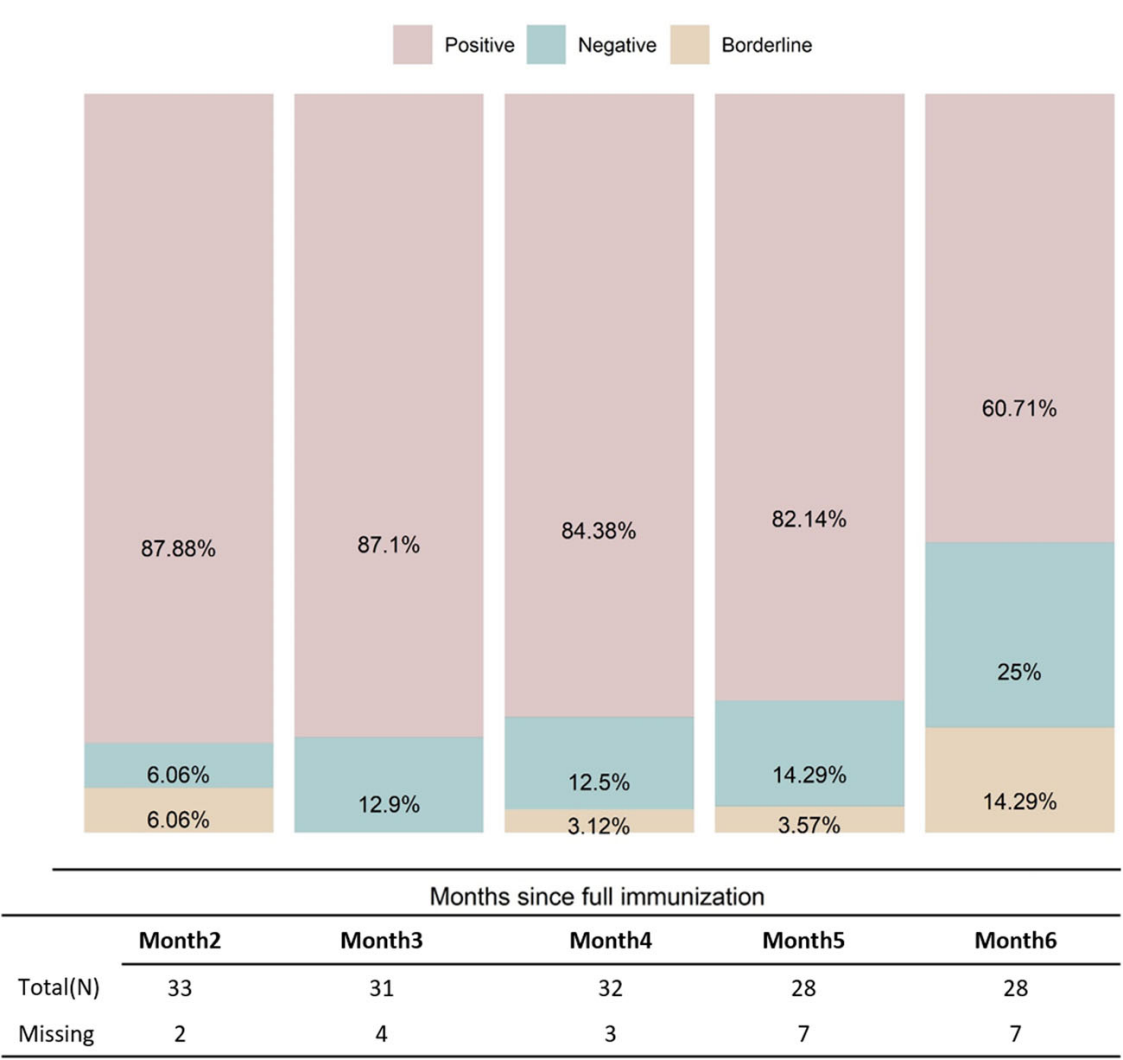
Percentage of persons who were positive, negative, and borderline based on antibody level by time point from a baseline sample collection of month ~2 post full vaccination to month ~6 post full vaccination. Percentages are calculated based on the total (*N*) of the table.

Based on the antibody level cutoffs provided by the manufacturer at 6 months post full immunization (positive, borderline, and negative), 61% (17/28) of patients maintained positive antibody levels, while 39% (11/28) had borderline or negative antibody levels (Figure 2). Additionally, the prediction of antibody level at month 10 post full vaccination demonstrated that more than 65% of the study population is anticipated to progress to borderline or negative antibody status (Figure 1).

## 4 DISCUSSION

The goal of the study was to define long‐term SARS‐CoV‐2 spike protein antibody response decay curves in a cohort of early‐vaccinated prevalent hemodialysis patients. The trajectory of long‐term IgG spike protein antibody decline and the association of antibody level with patient characteristics was analyzed. The data also confirm previously described findings showing lower rates of seroconversion in dialysis patients compared to the general population, as well as antibody level attenuation associated with immune suppression and advancing age.

A generally stable decline in IgG spike protein antibody levels from month to month regardless of subgroup or initial antibody peak was observed. This relatively stable decay rate suggests that the peak‐attained antibody level post vaccination is a predictive factor determining the duration of detectable IgG spike protein antibody levels. In fact, none of the previously infected patients is projected to lose detectable antibody levels through 10 months post full immunization (Figure 1B). Subjects with prior COVID‐19 infection developed higher antibody levels at initial measurement (Figure 1 and Supporting Information: Figure 2) and these remained significantly higher 6 months post full vaccination relative to infection‐naive patients (888.01 vs. 61.01 BAU/ml, respectively) (Table 1). The majority of the study cohort was infection naive. Thus, approximately 65% of subjects were projected to lose detectable IgG spike protein antibody at 10 months post full vaccination. This real‐world data through 6 months post full vaccination already demonstrates nearly 40% of antibody levels are at borderline or negative thresholds. While immune‐suppressed patients were expected to lose detectable antibody levels, the 40% of patients with borderline or negative antibody levels was a surprise.

Immunity and vaccine effectiveness are determined by many factors, not solely humoral components. However, there appears to be an inversely proportional relationship between antibody levels and symptomatic SARS‐CoV‐2 infection.^10^ Therefore, dialysis patients, highly vulnerable to SARS‐CoV‐2, may benefit from planned boosters at the population level. Data on response to boosters in hemodialysis patients is limited. A cohort of French dialysis patients demonstrates significant increases in spike protein IgG levels following the third dose of BNT162b2 given at a median of 50 days following a protocol second shot of BNT162b2.^11^

The study has limitations, which may limit generalizability. Notably, this was a small sample size and a nonrepresentative sample compared to overall US dialysis demographics. The small sample size precludes deeper analysis into clinical risk factors that affect the decline in antibody levels over time. Only the antibody response to BNT162b2 and not other COVID‐19 vaccines was reported. Lastly, although data have described a correlation between spike protein IgG levels and infection vulnerability, protective antibody levels have not been clearly determined. Thus, results should be cautiously interpreted. Strengths of the study include the long‐term nature, diverse comorbidities of the cohort, and the relatively complete data set allowing the development of antibody level trajectory curves.

In conclusion, long‐term IgG spike protein antibody decline rates in response to vaccination with BNT162b2 were determined. The declining antibody levels suggest that dialysis patients vaccinated with BNT162b2 will greatly benefit from receipt of a booster dose.

## AUTHOR CONTRIBUTIONS

**Eibhlin Goggins**: Conceptualization; data curation; investigation; writing – original draft; writing – review and editing. **Binu Sharma**: Formal analysis; methodology; software; visualization; writing – review and editing. **Jennie Z. Ma**: Formal analysis; methodology; software; writing – review and editing. **Jitendra Gautam**: Data curation; investigation; methodology; resources; writing – review and editing. **Brendan Bowman**: Conceptualization; funding acquisition; investigation; project administration; resources; supervision; validation; writing – review and editing.

## CONFLICT OF INTEREST

The authors declare no conflict of interest.

## ETHICS STATEMENT

This research proposal was reviewed and approved by the University of Virginia Institutional Review Board for Health Sciences Research (tracking number: HSR 210095).

## TRANSPARENCY STATEMENT

The lead author Eibhlin Goggins affirms that this manuscript is an honest, accurate, and transparent account of the study being reported; that no important aspects of the study have been omitted; and that any discrepancies from the study as planned (and, if relevant, registered) have been explained.

## Supporting information

Supporting information.

Supporting information.

Supporting information.

Supporting information.

## Data Availability

The data set used for this analysis is not publicly available. The data utilized was obtained from the Electronic Health Record and from the dialysis‐specific electronic medical record system, which is restricted to use by only authorized employees.
