# Supplementary material for: Long‐term humoral immunity decline in hemodialysis patients following severe acute respiratory syndrome coronavirus 2 vaccination: A cohort study
Source: Health Sci Rep. 2022 Oct 3;5(6):e854. doi: 10.1002/hsr2.854 (PMC9528757; doi:10.1002/hsr2.854)
Supplement: Supplementary file 1 — Supporting information. [file HSR2-5-e854-s002.docx]

**Supplemental Table 1:** Monthly Antibody Level (BAU/mL) by Clinical Characteristics

|  | **Month** | | | | | |
| --- | --- | --- | --- | --- | --- | --- |
|  | **2** | **3** | **4** | **5** | **6** |  |
| **Male** | 767.00 ± 790.53 | 518.12 ± 462.76 | 403.58 ± 534.15 | 312.10 ± 410.84 | 160.78 ± 264.79 |  |
| **Female** | 535.21 ± 775.07 | 466.32 ± 622.89 | 327.53 ± 468.21 | 290.37 ± 461.16 | 205.06 ± 367.58 |  |
| **Race** |  |  |  |  |  |  |
| African American | 824.36 ± 901.97 | 631.87 ± 6.92 | 477.76 ± 595.82 | 430.68 ± 519.17 | 258.11 ± 378.84 |  |
| White | 314.49 ± 404.72 | 258.81 ± 313.95 | 171.35 ± 211.71 | 119.32 ± 155.20 | 48.41 ± 69.01 |  |
| **Cancer History** | 494.29 ± 532.41 | 422.13 ± 405.78 | 273.55 ± 333.29 | 189.36 ± 222.65 | 82.46 ± 92.86 |  |
| **Comorbidities** |  |  |  |  |  |  |
| DM | 523.41 ± 646.69 | 424.73 ± 474.77 | 280.11 ± 368.19 | 230.04 ± 354.63 | 138.37 ± 259.05 |  |
| CVA | 366.29 ± 396.16 | 252.27 ± 243.10 | 163.70 ± 181.35 | 165.91 ± 153.43 | 66.26 ± 60.87 |  |
| CHF | 787.46 ± 881.98 | 558.36 ± 534.05 | 415.01 ± 568.11 | 324.62 ± 480.66 | 214.83 ± 360.49 |  |
| MI | 769.91 ± 736.04 | 516.97 ± 545.10 | 354.42 ± 467.83 | 322.69 ± 480.51 | 208.90 ± 369.13 |  |
| Obesity (BMI>30) | 640.26 ± 920.00 | 465.06 ± 619.91 | 355.98 ± 367.19 | 323.43 ± 354.63 | 230.66 ± 259.05 |  |
