# Supplementary figures and images for: Long‐term humoral immunity decline in hemodialysis patients following severe acute respiratory syndrome coronavirus 2 vaccination: A cohort study
Source: Health Sci Rep. 2022 Oct 3;5(6):e854. doi: 10.1002/hsr2.854 (PMC9528757; doi:10.1002/hsr2.854)

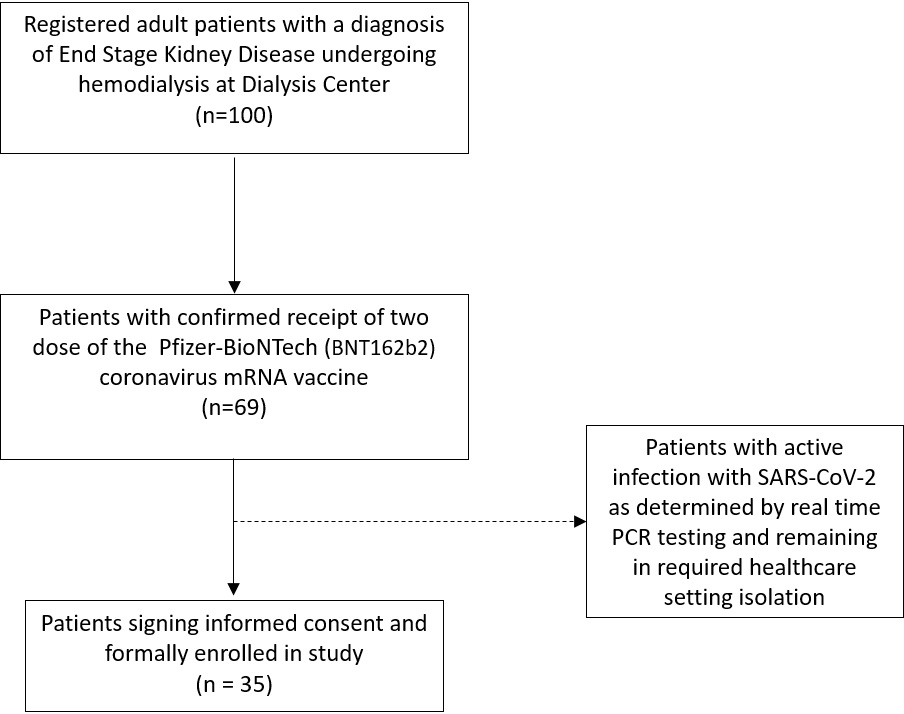

Supplement: Supplementary file 2 — Supporting information. [file HSR2-5-e854-s001.jpg]

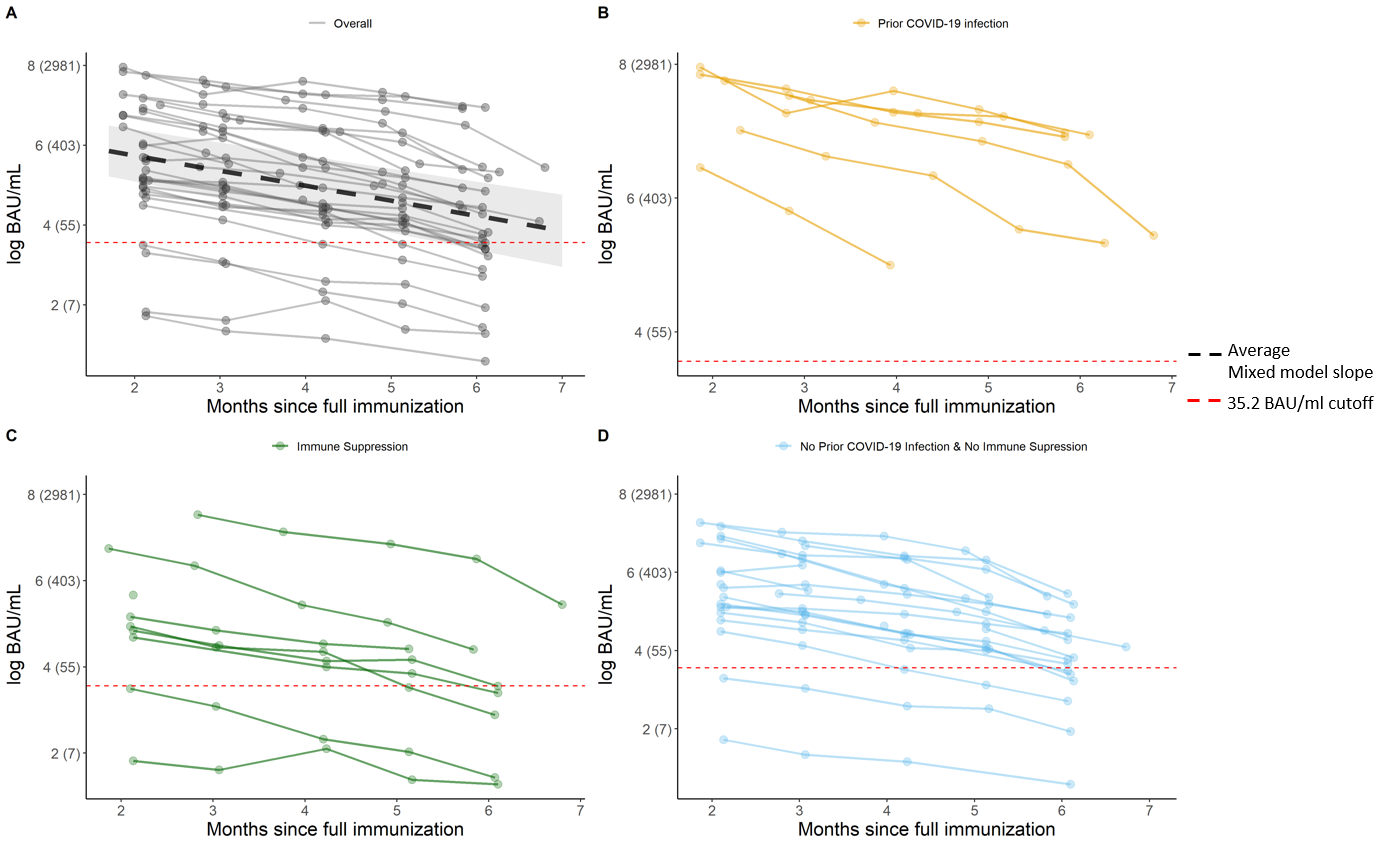

Supplement: Supplementary file 3 — Supporting information. [file HSR2-5-e854-s004.tif]
