# Supplementary material for: Long‐term humoral immunity decline in hemodialysis patients following severe acute respiratory syndrome coronavirus 2 vaccination: A cohort study
Source: Health Sci Rep. 2022 Oct 3;5(6):e854. doi: 10.1002/hsr2.854 (PMC9528757; doi:10.1002/hsr2.854)
Supplement: Supplementary file 4 — Supporting information. [file HSR2-5-e854-s003.docx]

**Supplemental Figure 1:** Study Participant Inclusion Flow Chart

**Supplemental Figure 2:** Spaghetti plot for observed antibody levels of SARS-CoV-2 over time from month ~2 to month ~6 post full immunization (i.e., 14 days after second dose of vaccination) in (A) Overall sample, (B) Prior COVID-19 infection group, (C) Immune suppression group, (D) Naïve group, No prior infection, and No immune suppression group.

Antibody levels are presented in log scale and actual values are shown inside parentheses. The cutoff for borderline/negative antibody level was defined according to manufacturer i.e. 35.2 BAU/mL (red dashed line). The estimated unadjusted slope by linear mixed model including all 35 subjects is shown in A (thick black dashed line).
